# Clinical, epidemiological, and laboratory analysis of hospitalized and fatal COVID-19 cases in the first fully vaccinated municipality in Northeast Brazil

**DOI:** 10.1590/S1678-9946202567050

**Published:** 2025-08-18

**Authors:** Lourrany Borges Costa, Arina Peixoto Nobre, Maria Eduarda Soares dos Santos, Luís Arthur Brasil Gadelha Farias, Magda Moura de Almeida, Antonia Luciana Souza Bekman, Silvana Soares de Souza, Francisca Kalline de Almeida Barreto, Ana Carolina Barjud Marques Máximo, Debora Bezerra Silva, Roberto Wagner Júnior Freire de Freitas, Luciano Pamplona de Góes Cavalcanti

**Affiliations:** 1 Universidade Federal do Ceará, Programa de Pós-Graduação em Saúde Pública, Fortaleza, Ceará, Brazil; 2 Universidade de Fortaleza, Faculdade de Medicina, Fortaleza, Ceará, Brazil; 3 Centro Universitário Christus, Faculdade de Medicina, Fortaleza, Ceará, Brazil; 4 Secretaria Municipal de Saúde de Guaramiranga, Guaramiranga, Ceará, Brazil; 5 Laboratório Central de Saúde Pública do Ceará, Fortaleza, Ceará, Brazil; 6 Fundação Oswaldo Cruz, Eusébio, Ceará, Brazil

**Keywords:** COVID-19 vaccines, Vaccination coverage, Case series, Hospitalization

## Abstract

Guaramiranga, Ceara State, Brazil, a tourist city 105.5 km from the capital Fortaleza, was a pilot site for vaccinating 100% of its 4,002 adult population with the first dose against COVID-19. The city received 3,328 CoronaVac, 1,685 AstraZeneca, and 174 Pfizer–BioNTech doses (5,187 in total). Vaccination with the first dose occurred from January 20, 2021 to April 1, 2022. This study analyzed hospitalized patients’ epidemiological, clinical, and laboratory characteristics and deaths from COVID-19 in Guaramiranga from March 2020 to December 2022. In total, nine cases required hospitalization, seven of which resulted in death. Patients’ median age at death equaled 87 (64-95) years. Only one was male, and five had incomplete vaccination schedules before their hospitalization. Hypertension and diabetes configured the most frequent comorbidities, whereas common symptoms at admission included fever (four cases), cough (four cases), and dyspnea (five cases). Of these nine patients, 4 received anticoagulants and corticosteroids; 6, oxygen supplementation; 2, oseltamivir; and 3, empirical antibiotic for pneumonia. The mean interval between the last vaccine dose and hospitalization totaled 128 days (28–326; SD 137). These findings highlight incomplete vaccination, a prolonged interval since last doses, comorbidities, and advanced age as key factors associated with severe cases and deaths and emphasize the protective role of high vaccination coverage.

## INTRODUCTION

COVID-19, caused by the β-coronavirus SARS-CoV-2, is an infectious respiratory disease that can bind to human cells via the angiotensin-converting enzyme 2 (ACE2) receptor. Viral replication starts in the nasal cavity and throat, spreading to the lower respiratory tract and gastrointestinal mucosa, with ACE2-expressing tissues—such as the nasal mucosa, lungs, heart, kidneys, and stomach—being particularly vulnerable. Severe complications include severe acute respiratory syndrome (SARS), which can lead to hospitalization and death^1,2^.

The rapid spread of cases prompted the World Health Organization to declare a pandemic in March 2020^3^. Currently, Brazil ranks sixth worldwide in cases (37.5 mi) and second in deaths (702k)^4^. Ceara, a state in northeastern Brazil, confirmed its first COVID-19 cases on March 15, 2020. Data from various government sources during the first 45 days of the epidemic in the state showed a 71.3% occupancy rate for hospital ward beds and an 80.5% occupancy in intensive care unit (ICU) beds^5^.

The impact of the COVID-19 pandemic on small towns differed significantly from that in large urban centers, highlighting structural inequalities and differences in social isolation patterns and access to public healthcare. Large municipalities showed sharper declines in urban mobility, greater reliance on non-pharmaceutical interventions to control virus transmission, and slower recovery rates, even with high vaccination coverage. In contrast, small municipalities experienced more subtle impacts on social mobility, primarily affecting vulnerable groups such as older adults. These differences may have stemmed from the lower population density and less dynamic lifestyle in small municipalities, which helped mitigate some of the direct impacts of the pandemic^6,7^. However, in Brazilian municipalities in regions such as Minas Gerais State, the limited availability of ICU beds configured a determining factor to their higher mortality rates, underscoring the structural limitations of these places in facing the health crisis^8^.

This study analyzes the epidemiological, clinical, and laboratory characteristics and outcomes of a series of hospitalized cases and deaths in patients with COVID-19 from June 2020 to December 2022 in Guaramiranga, Ceara State. The municipality’s primary public health facility is the hospital, which has six inpatient adult beds. This study is part of a more extensive investigation approved by the Research Ethics Committee of the Christus University Center (CAAE No. 51252221.3.0000.5049) and conducted in accordance with the 1964 Declaration of Helsinki and its subsequent amendments.

## MATERIALS AND METHODS

In this case series, data were collected from September 2021 to December 2022 by a retrospective review of medical records and death certificates at the Municipal Hospital of Guaramiranga. Inclusion criteria included patients hospitalized during the study period with confirmed COVID-19 diagnoses established via RT-PCR or rapid antigen tests since the onset of the pandemic in March 2020. The collected data referred to patients’ demographics (age, sex), comorbidities, vaccination status (including vaccine type and dates of doses), symptoms at admission, clinical management (medications, oxygen therapy, mechanical ventilation), and outcomes (hospital discharge or death). The interval between their last vaccine dose and hospitalization was also calculated. Patients’ characteristics and outcomes were summarized by descriptive statistics. Results were analyzed for the factors associated with severe disease and mortality, emphasizing the role of vaccination, comorbidities, and age in disease progression.

The Guaramiranga municipality in Ceara State lies in a mountainous region 105.5 km from the state capital, Fortaleza. It had an estimated population of 5,193 inhabitants, of whom 4,002 are adults aged 18 or older according to the 2019 census^9^. In 2021, Guaramiranga received 5,187 COVID-19 vaccines for the first dose (D1), including 3,328 doses of CoronaVac from the Sinovac/Butantan manufacturer, 1,685 of AstraZeneca/Oxford/Fiocruz, and 174 of Pfizer–BioNTech (Comirnaty). D1 was administered from January 20, 2021 up to April 1, 2022. The second dose (D2) was administered from February 18, 2021 (28 days after D1) up to July 6, 2022. It was the first municipality in Ceara to vaccinate 100% of its registered population. Since the beginning of the pandemic in March 2020 to February 2022, 4,663 suspected cases of the disease were reported in Guaramiranga, of which 1,565 were confirmed and six resulted in death^10^.

The healthcare system in Guaramiranga includes only three primary healthcare units and a small-sized hospital with six inpatient adult beds. SARS-CoV-2 diagnosis followed the municipal contingency plan (March 2020), using RT-PCR and rapid antigen tests. Mild cases were managed in primary care, whereas hospitalized patients received care at the municipal hospital. Severe cases requiring high-complexity care were referred to hospitals in Fortaleza or neighboring cities, as per an agreement with the Ceara State Health Department^10^.

## RESULTS

### General aspects of hospitalized cases

Only nine confirmed cases were hospitalized out of 1,565. The median age of death totaled 87 (64-95) years. Only one case involved a man, which occurred before the start of the vaccination campaign. Additionally, four out of those nine patients had failed to complete their vaccination schedule before hospitalization due to COVID-19 (Table 1). No patients underwent mechanical ventilation as the small facility of the Municipal Hospital of Guaramiranga lacks a ventilatory support infrastructure. However, six cases required supplemental oxygen therapy during hospitalization or while awaiting transfer, which was administered via nasal catheter or reservoir-equipped non-rebreather face mask.

**Table 1:** Clinical and epidemiological characteristics of hospitalized COVID-19 cases in Guaramiranga, Ceara State, Brazil (June 2020 to December 2022).

Case		1	2	3	4	5	6	7	8	9
**Age (years)**		85	90	87	64	95	90	54	82	64
**Sex**		M	F	F	F	F	F	F	F	F
**Comorbidities**		-	T2DM and other unspecified chronic cardiac and respiratory diseases	-	HTN, T2DM, and unspecified chronic heart disease	T2DM, unspecified chronic heart disease, and advanced dementia	HTN, T2DM, and dementia	Dyslipidemia and unspecified mental disorder	Ex-smoker, COPD using home oxygen	-
**Vaccination status**		Unvaccinated	Unvaccinated	1 dose of CoronaVac and 1 dose of AstraZeneca	1 dose of AstraZeneca	2 doses of AstraZeneca	2 doses of CoronaVac	Unvaccinated	1 dose of CoronaVac and 1 dose of AstraZeneca	Unvaccinated
**Date of admission or onset of symptoms or diagnosis**		Jun 20, 2020	Feb 2, 2021	Feb 3, 2021	Jun 4, 2021	Jun 6, 2021	Jan 27, 2022	Jan 14, 2022	May 6, 2022	-
**Days between last vaccine dose and admission**		-	-	217	30	37	326	-	28	-
**Outcome**		Death	Death	Death	Death	Death	Death	Hospital discharge	Hospital discharge	Death
**Time (in days) until outcome**		5	5	0	12	10	0	3	-	14
**Symptoms on admission/ Symptoms on the day of outcome**		Pneumonia/AKI and CPA	Dyspnea/		Low	Fever, cough and dyspnea/ SARS and CPA		Fever, cough, dyspnea, and cognitive impairment/	Cough/	Fever, cough, and dyspnea/
					SpO_2_ and hyperglycemia /SARS and CPA					
**Signs/examinations on admission**	**Blood pressure (mmHg)**		-	150x90	120x80	-	100x50	90x60	130x80	140x90
	**Heart rate (bpm)**		-	118	79	-	83	60	-	-
	**Respiratory rate (rrpm)**		-	37	28	-		-	30	-
	**SpO**_**2**_ **(%)**		-	90	88	-	95	92	71	84
	**Capillary blood glucose (mg/ dL)**		-	109	266	-	105	131	-	151
	**Temperature (** ^**o**^ **C)**		-	-	-	-	36	-	-	-
	**COVID-19 Diagnostic Test**		RT-PCR		Rapid antigen research test	RT-PCR	RT-PCR	Rapid antigen research test	-	Rapid antigen research test
	**Hemoglobin**		-	-	-	-	-	11.6	13.7	-
	**Hematocrit**		-	-	-	-	-	34.9	45.1	-
	**Leukocytes**		-	-	-	-	-	3700	10700	-
	**Lymphocytes**		-	-	-	-	-	1221	856	-
	**Platelets**		-	-	-	-	-	189000	235000	-
	**LDH**		-	-	-	-	-	554	-	-
	**TGO**		-	-	-	-	-	20	24	-
	**TGP**		-	-	-	-	-	16	18	-
	**Urea**		-	-	-	-	-	17	30	-
	**Creatinine**		-	-	-	-	-	0.85	1.1	-
	**D-dimer**		-	-	-	-	-	-	-	202.97
	**Troponin**		-	-	-	-	-	-	-	< 0.1
	**Urine**		Bacteriuria	-	-	-	-	-	-	-
**Main therapy instituted on admission**		Transfer to ICU	-	Hydrocortisone, ceftriaxone, ipratropium bromide	Dexamethasone, ceftriaxone, azithromycin, enoxaparin, salbutamol, and supplemental oxygen	-	-	Hydrocortisone, dexamethasone, enoxaparin, and supplemental oxygen	Transfer to ICU	Dexamethasone, ceftriaxone, enoxaparin, supplemental oxygen. Transfer to ICU

AKI = Acute kidney injury; CPA = Cardiopulmonary arrest; COPD = Chronic obstructive pulmonary disease; D1/D2 = First dose/second dose of COVID-19 vaccine; F = female; HTN = Hypertension; LDH = Lactate dehydrogenase; M = male; RT-PCR = Reverse transcription polymerase chain reaction; SARS = Severe acute respiratory syndrome; SpO_2_ = Peripheral oxygen saturation; T2DM = Type 2 diabetes mellitus; TGO = Aspartate aminotransferase; TGP = Alanine aminotransferase; ICU = Intensive care unit.

## CASE REPORTS

### Case 1

The 85-year-old male patient had no reported comorbidities. Symptoms began on June 20, 2020, with pneumonia reported on admission. He was admitted to the Hospital of Guaramiranga and later transferred to a reference hospital in Fortaleza. According to the death certificate, he developed COVID-19 pneumonia and an acute renal injury, leading to death five days later, on June 25, 2020.

### Case 2

The 90-year-old female patient had comorbidities, including type 2 diabetes mellitus (T2DM) and other unspecified chronic cardiac and respiratory diseases. She refused to receive the vaccine. She was admitted to the hospital in Guaramiranga due to T2DM decompensation. She was later referred to a reference hospital in Fortaleza: Dr. Carlos Alberto Studart Gomes Messejana Hospital, at which she underwent RT-PCR testing for SARS-CoV-2 viral RNA due to the onset of dyspnea symptoms on January 17, 2021, and suspected COVID-19. The initial RT-PCR result was undetectable. On February 2, 2021, the RT-PCR test was repeated, obtaining a detectable result. The patient developed SARS and cardio-pulmonary arrest, leading to death five days later, on February 7, 2021.

### Case 3

The 87-year-old woman reported no comorbidities. She received a D1 vaccine of AstraZeneca/Oxford/Fiocruz on February 1, 2021 and a D2 of CoronaVac on April 30, 2021. Symptoms began on February 3, 2021, 217 days after D2. She was admitted with respiratory distress, hypoxia, tachypnea, and hypertension (HTN). Pulmonary auscultation showed generalized wheeze and right-sided rales. RT-PCR confirmed COVID-19. She received hydrocortisone, ceftriaxone, intravenous hydration, nebulization with ipratropium bromide, analgesics, antipyretics, and antiemetics. An oxygen reservoir-equipped non-rebreather face mask supplied the patient with 15 L/min. Laboratory urinalysis showed nitrites, urobilinogen, leukocytes, and severe bacteriuria. The patient had heavy sweating, hypotension (86/57 mmHg), tachypnea (37 breaths per minute), tachycardia (118 beats per min), and hypothermia (35 °C) 9 hours after admission. Physical examination showed pallor, cold extremities, absent right lung vesicular breath sounds, and a bloated belly. The patient was recommended for transfer to a reference hospital in Fortaleza, which the family declined. According to medical records, the hospital team and family met to discuss the gravity of the case and the decision not to use invasive measures. Then, 20 minutes later, the patient died of SARS on the same day of her admission.

### Case 4

A 64-year-old female patient had HTN, T2DM, and chronic cardiac diseases. She received one AstraZeneca/Oxford/Fiocruz vaccine dose on May 5, 2021. She presented with fever, cough, and dyspnea. A positive rapid antigen test on May 28, 2021, diagnosed her with COVID-19 (23 days after D1). She was hospitalized on June 4, 2021 (30 days after D1), with an oxygen saturation (SpO_2_) of 88% and capillary blood glucose of 266 mg/dL. To improve oxygenation, she received supplemental oxygen via nasal cannula at a flow rate of 5 L/min. Additionally, treatment began with dexamethasone, ceftriaxone, azithromycin, enoxaparin, salbutamol, and symptomatic medications. Transfer to a hospital in a neighboring city was requested. Despite treatment, the patient developed SARS and died 12 days later, on June 16, 2021.

### Case 5

A 95-year-old female patient had T2DM, chronic cardiac disease, and advanced dementia. She received two doses of AstraZeneca/Oxford/Fiocruz, the second of which was administered 37 days before her first hospitalization. Her first hospitalization began from June 6 to June 15, 2021, when she was admitted with fever, cough, and dyspnea. An RT-PCR test conducted on June 7, 2021, was positive for SARS-CoV-2. The second hospitalization began on June 16, 2021, during which SARS was confirmed the following day, with SpO_2_ at 76% despite oxygen support via a non-rebreather mask (15 L/min). According to medical records, the decision to neither intubate nor perform invasive measures, including transfer to another health facility, occurred in consultation with her family. The patient died on the same day.

### Case 6

A 90-year-old female patient had HTN and unspecified dementia. She had received two doses of the CoronaVac vaccine, with the D2 administered 326 days before admission. Unspecified respiratory issues led to hospitalization 2 days later. A positive RT-PCR test 8 days after admission confirmed COVID-19. The patient received oseltamivir, aminophylline, metoprolol, intravenous hydration, tramadol, intravenous glucose, and head-of-bed elevation. Her family declined intubation and transfer to another facility. She died on the same day of hospital admission.

### Case 7

A 54-year-old female patient had dyslipidemia and an unspecified mental disorder. She refused to take the vaccine at the beginning of the campaign. She presented with fever, cough, dyspnea, and cognitive alterations on January 14, 2022. A rapid antigen test confirmed COVID-19. On examination, she had hypotension and a SpO_2_ of 92% on room air. Supplemental oxygen was administered via a nasal cannula at 2 L/min. Treatment included hydrocortisone, dexamethasone, enoxaparin, symptomatic medications, and her ongoing psychotropic medications. Chest X-ray showed the characteristic bilateral infiltrates. The patient improved clinically and was discharged on February 17, 2022, 3 days later, and accepted the first dose of the AstraZeneca/Oxford/Fiocruz vaccine on July 6, 2022.

### Case 8

An 82-year-old female patient, a former smoker, had Alzheimer’s disease and chronic obstructive pulmonary disease (COPD), receiving home oxygen therapy. She received two doses of COVID-19 vaccines (CoronaVac and AstraZeneca), with hospitalization occurring 28 days after D2. RT-PCR confirmed COVID-19. She was hospitalized on May 6, 2022, with a 3-day history of cough and dyspnea. When admitted, she had tachypnea, reduced vesicular breath sounds, and diffuse expiratory wheezing on pulmonary auscultation with a 71% SpO_2_ level. Hydrocortisone, beclomethasone, ceftriaxone, azithromycin, enoxaparin, oseltamivir, salbutamol, ipratropium, and intravenous hydration were administered. A non-rebreather mask provided her with 7 L/min of oxygen. Chest X-ray showed diffuse infiltration. The next day, the patient became unconscious and was taken to the resuscitation room to receive furosemide, morphine, hydralazine, nitroglycerin, norepinephrine, and dobutamine. She was then transferred to the ICU of a neighboring city, where she improved and was discharged.

### Case 9

A 63-year-old female patient, a former smoker, had HTN and T2DM. She refused to take the vaccine. She presented fever, cough, and dyspnea after 10 days of symptomatic COVID-19 infection (confirmed via rapid antigen test). She sought assistance, and on admission, her SpO_2_ was 84%. Treatment included dexamethasone, unfractionated heparin, ceftriaxone, and oxygen via a reservoir mask. After 2 days of hospitalization, her cough and dyspnea worsened, and she experienced persistent fever. On the fifth day, SpO_2_ levels dropped despite a non-rebreather mask at 15 L/min, requiring transfer to a tertiary hospital ICU. The patient died 14 days after admission.

## DISCUSSION

Although this study involves a small series of severe COVID-19 cases and deaths in the first municipality in northeastern Brazil to achieve 100% adult vaccination, its data suggest that completing the vaccination schedule likely reduced the chances of progression to severe disease and death. The mean age of severe cases in our study was 79 years (range: 54–95; SD: 14.5), whereas the mean age of death was 82. Additionally, all severe cases occurred in older adults with comorbidities. Most rejected vaccination at the time of the campaign, and the others had an incomplete vaccination schedule for COVID-19.

Fever, cough, and dyspnea configured the primary admission complaints in Guaramiranga, in line with findings from previous studies^1,2^. Moreover, six of these nine cases had comorbidities, including hypertension, diabetes, obesity, and chronic renal disease, conditions that promote persistent inflammation, vascular injury, and impaired tissue repair, thereby exacerbating COVID-19 severity^11^. These findings agree with a Chinese study with 1,099 patients, 44% of whom presented with fever at admission, increasing to 89% during hospitalization^1^. Age configured the primary risk factor for severe disease, particularly in those aged over 65 years.

Several epidemiological studies support these findings: seven-day hospitalization medians (range: 3–9) after symptom onset; hospitalized patients with a median age of 47-73 years (74-86% were aged over 50 years) and comorbidities such as HTN (48%-57%), DM (17%-34%), cardiovascular diseases (21%-28%), chronic lung diseases (4%-10%), chronic kidney diseases (3%-13%), cancer (6%-8%), and chronic liver diseases (<5%). Approximately 35% of patients received treatment in ICUs (most often for respiratory failure) and 91% required invasive mechanical ventilation^12^.

The COVID-19 pandemic has spurred significant efforts to develop therapeutic strategies. Several antiviral drugs and monoclonal antibodies have been approved and commercialized for the treatment of COVID-19. At the time, four of the nine chosen hospitalized cases received enoxaparin, dexamethasone, hydrocortisone, oxygen supplementation, or mechanical breathing. These methods follow the Brazilian Ministry of Health (MH) COVID-19 management guidelines at that time^13^.

In 2020, the United Kingdom began a controlled trial involving approximately 6,425 hospitalized patients, of whom 2,104 received dexamethasone treatment. After 28 days, dexamethasone reduced mortality in patients who depended on invasive mechanical ventilation or oxygen. Another randomized clinical trial found that hospitalized COVID-19 patients on oxygen therapy who received large doses of dexamethasone had better clinical symptoms within 11 days^14^. These findings corroborate the MH recommendations and the management of cases reported in Guaramiranga.

Only two cases in Guaramiranga received oseltamivir. During the COVID-19 pandemic, the MH recommended prioritizing its use within 48 h of symptom onset for high-risk cases of SARS and ILI^15^. Evidence supporting the use of oseltamivir to treat COVID-19 remains inconsistent^16,17^. A meta-analysis of observational studies from Indonesia, South Korea, Iran, and China (2020-2021) found no significant survival benefit but reported a reduced hospitalization stay^18^. Conversely, a large retrospective cohort study in Brazil (2020-2023) suggested a modest reduction in mortality, particularly in ICU patients on ventilation^17^. Given these conflicting findings, prospective studies are needed to elucidate the role of oseltamivir in COVID-19 treatment.

Subsequent advancements in treatment protocols approved additional antiviral therapies, such as remdesivir and Paxlovid (nirmatrelvir/ritonavir) for high-risk patients^19-22^. From 2023, monoclonal antibodies such as tocilizumab and immunomodulators such as baricitinib were incorporated to reduce the need for mechanical ventilation in severe cases^23,24^. Additionally, the MH and the American Society of Hematology recommend prophylactic-intensity anticoagulation for acutely ill COVID-19 patients, with therapeutic-intensity anticoagulation considered for select cases based on individual risk assessment^25^.

In total, three out of nine Guaramiranga hospitalized cases received empirical medications for pneumonia (either azithromycin, ceftriaxone, or both) for suspected cases of coinfection, as recommended by the MH^13^. The reported cases partially adhere to the Brazilian Society of Pulmonology and Phthisiology Community-Acquired Pneumonia Guidelines, which recommend respiratory fluoroquinolones or a β-lactam combined with a macrolide for hospitalized patients^26^. Similarly, the American Thoracic Society and the Infectious Diseases Society of America suggest a β-lactam plus a macrolide or fluoroquinolone for severe community-acquired pneumonia without risk factors for MRSA or *Pseudomonas aeruginosa* ^27^.

Note that Brazil widely used azithromycin for mild COVID-19 cases early in the pandemic^28^ despite a lack of clinical benefit confirmed by large randomized trials^14,29^. Similarly, multiple other medications have shown no proven efficacy against COVID-19, reinforcing the need to avoid off-label use without scientific evidence^13,30^.

We found four unvaccinated patients, three of whom died. A 54-year-old lady was discharged unvaccinated. She was the youngest and only released patient of the nine hospitalized instances. In total, four vaccinated patients completed a two-dose regimen: CoronaVac, AstraZeneca, and two other manufacturers. The mean between the last vaccine dose and hospitalization totaled 128 days (range: 28–326; SD: 137). Thus, long-term infections after vaccination may explain hospitalizations. SARS-CoV-2 immunity declines from the first month following vaccination to the sixth month, when it may no longer be effective^31^. The vaccine, number of doses, hybrid immunity, age, and immunocompromised status affect immunity duration^32,33^.

A Hong Kong study with 2,780 vaccinated individuals, including 799 CoronaVac recipients, assessed neutralizing antibody strength and durability after two doses. The mean level of neutralizing antibodies 14 to 42 days after vaccination totaled 53.7% (95% CI: 51.2–56.3) but 23% had levels below 30%. Levels rose to 51% over 60 days and to 84% from 120 to 180 days^34^. AstraZeneca creates humoral and T-cell immunity. Antibody levels reach their highest point 28 days after the second dose and slowly drop over the next six months. T-cell responses emerge 14-22 days after the first dose but show little increase post-second dose, persisting for 90 days^32^.

Thus, at least three reported cases received inadequate protection against COVID-19 despite their vaccination. These data underscore the importance of health managers considering specific evidence of vaccine efficacy and effectiveness when deciding on the need and timing for additional booster doses.

According to Guaramiranga City Hall data, the municipality had 1,499 confirmed cases of COVID-19 from July 2020 to February 23, 2022, with six deaths and four hospitalized patients who also died^10^. Using these data and applying the Pandemic Severity Assessment Framework^35^, the impact of the disease in Guaramiranga during the analyzed period would be classified as having a low overall impact on the healthcare system given its case fatality rate (0.4%) and hospitalization proportion (0.27%). However, it would be categorized as posing a moderate risk of clinical severity due to its high hospital mortality rate (100%), highlighting significant structural vulnerabilities in the local healthcare system. These findings emphasize the need to prioritize preventive measures, such as COVID-19 vaccination in small towns to mitigate healthcare burdens and reduce severe outcomes.

This study has some limitations. Data were retrospectively collected from medical records and death certificates, which may have led to inaccuracies and omissions. The case series in this study may have excluded other cases that could have occurred, especially if patients had decided to seek assistance directly in the state capital, Fortaleza. While this scenario is unlikely, this research is unable to definitively exclude it.

## CONCLUSION

Patients’ profile showed hospitalizations and fatal outcomes due to COVID-19 in individuals with comorbidities, older adults, and unvaccinated individuals or those with incomplete vaccination schedules. This reinforces the finding that these populations show greater vulnerability to the disease. Thus, prevention and treatment strategies should prioritize them. Vaccination coverage likely contributed to the small number of severe cases and fatal outcomes in this study.
